# Outbreak-driven differences in the microbiome composition and diversity of two cassava whitefly *Bemisia tabaci* mitotypes SSA1-SG1 and SSA1-SG2

**DOI:** 10.3389/fmicb.2025.1597836

**Published:** 2025-07-11

**Authors:** Hajar El Hamss, Hadija M. Ally, Hélène Delatte, Christopher A. Omongo, John Colvin, M. N. Maruthi

**Affiliations:** ^1^Natural Resources Institute, University of Greenwich, Kent, United Kingdom; ^2^Phytopathology Unit, Department of Plant Protection, Ecole Nationale d’Agriculture de Meknès, Meknes, Morocco; ^3^Tanzania Agricultural Research Institute Ukiriguru, Mwanza, Tanzania; ^4^CIRAD, UMR PVBMT, Saint Pierre, France; ^5^Root Crops Programme, National Crops Resource Research Institute (RCP-NaCRRI), Kampala, Uganda

**Keywords:** whitefly, SSA1-SG1, SSA1-SG2, *Bemisia tabaci*, mtCO1, 16S rDNA, microbiome, Uganda

## Abstract

Since the 1990s, outbreaking populations of the whitefly *Bemisia tabaci* species complex (Sub-Saharan Africa 1 and 2) have heavily infested cassava in Uganda and eastern Africa. These superabundant SSA1 whiteflies from outbreaking areas carry microbiomes that might influence their fitness. Nonetheless, the factors contributing to the surge of these populations and their connection to the whitefly microbiome remain uncertain. To explore microbiome structure, diversity, and potential contributions to outbreaks of *B. tabaci* SSA1 species, we performed 16S rDNA amplicon sequencing. Endosymbionts (excluding *Portiera*) and the meta-microbiome were analyzed separately across 56 SSA 1 samples identified using a partial fragment of the mtCOI gene from 8 sites (32 outbreakings and 24 non-outbreakings). Two mitochondrial profiles were obtained within the samples named here as mitotypes SSA1-SG1 and SSA1-SG2. We investigated microbiome differences at two levels: (i) between two mitochondrial mitotypes, SSA1-SG1 and SSA1-SG2, and (ii) between outbreaking and non-outbreaking whitefly populations. Our results showed that the two mitotypes exhibited significantly different endosymbiont diversity (*p* < 0.0001), structures (*p* < 0.01, determined by ADONIS and Capscale), and co-occurrence networks. At the population level, significant differences in microbiome diversity were observed between outbreaking and non-outbreaking populations (Simpson index: *p* = 0.007; Shannon index: *p* = 0.006), with outbreaking populations showing reduced microbial diversity. Community structure also differed significantly (*p* = 0.001), as revealed by ADONIS and Capscale analyses using Bray–Curtis metrics. Outbreaking SSA1-SG1 whiteflies showed the highest microbial richness (mean = 63 ASVs), compared to an overall average of 45 ASVs across all samples. Co-occurrence patterns were highly structured, indicating non-random microbial interactions and shifts. Overall, our findings highlight the microbiome as a key factor in local invasions and epidemic emergence. Future research should focus on identifying specific bacterial contributors to better understand their role in outbreak dynamics.

## Introduction

The group of cryptic species of the whitefly, *Bemisia tabaci* (Hemiptera: Aleyrodidae) comprises more than 40 whitefly species (Elfekih et al., 2021; Campbell et al., 2023). In Uganda, 13 *B. tabaci* species have been identified infesting cassava, Sub-Saharan Africa (SSA), from 1 to 13 (SSA1-13). Five of these species (SSA9 to SSA13) were recently discovered based on mitochondrial cytochrome c oxidase Subunit 1 (mtCO1) analysis (Mugerwa et al., 2021a; Mugerwa et al., 2021b). Additionally, other *B. tabaci* species, including East Africa 1 (EA1), Indian Ocean (IO), and MED, have also been reported on cassava, albeit in low numbers (Mugerwa et al., 2021a). These different species within the *B. tabaci* complex can exhibit variations in their biology, behavior, host preferences, and ability to transmit plant viruses (Omondi et al., 2005; Civolani et al., 2014; Ning et al., 2015). Based on mtCO1 analysis, SSA1 was further subdivided into sub-groups, ranging from SSA1-SG1 to -SG5, also referred to as mitotypes. The SG1 and SG2 mitotypes were recently shown to belong to the same *B. tabaci* SSA1 species and renamed SSA1 SG1USG2 (Campbell et al., 2023).

*Bemisia tabaci* SSA1 SG1USG2 is an important pest affecting cassava and other crops in Uganda and other countries, causing damage by feeding on plant sap and reducing growth, yield, and crop quality (Kalyebi et al., 2018). Moreover, it is a vector for plant viruses causing economically important cassava mosaic disease (CMD) and cassava brown streak disease (CBSD) epidemics in Uganda (Maruthi et al., 2002; Colvin et al., 2006; Maruthi et al., 2017). In this work, we will refer to them as SSA1, SSA1-SG1, and SSA1-SG2 to denote the two mitotypes belonging to SSA1.

Outbreaks, defined as the presence of more than 100 whiteflies on the top five leaves of cassava plants, have persisted in Uganda since the 1990s (Legg et al., 2002; Legg et al., 2014; Macfadyen et al., 2018), impacting both small-scale and large-scale cassava production. Various factors have been suggested as drivers of increasing whitefly infestations, including monoculture cropping (Macfadyen et al., 2018), as well as the mutually beneficial interaction between cassava mosaic viruses and whiteflies or the enhanced genetic fitness of certain whiteflies on cassava (Legg et al., 2002).

*Bemisia tabaci* species carry primary and secondary endosymbionts, and the presence and dynamics of these endosymbionts can vary based on factors like spatiotemporal changes (El Hamss et al., 2022), life stage (El Hamss et al., 2024), and/or the host plant (Maruyama et al., 2020).

*Bemisia tabaci* species harbor endosymbiotic bacteria, which reside in the body cavity, haemolymph, or intracellularly in specialized cells called bacteriocytes (Ghosh et al., 2018). Endosymbionts can enhance the fitness of *B. tabaci* by improving food supply through the synthesis of essential nutrients, such as carotenoids (Sloan and Moran, 2012). Endosymbionts may also protect against natural enemies by increasing resistance to parasitoids or predators (Giorgini et al., 2023). *Portiera aleyrodidarum*, is a primary endosymbiont present in all *B. tabaci* species, including the SSA ones, and which supplements their amino acid-deficient diets. Secondary endosymbionts, such as *Rickettsia*, *Wolbachia*, and *Arsenophonus*, are present in SSA1 either in single or mixed infections (Ghosh et al., 2015). While *Hamiltonella*, *Cardinium*, and *Fritschea* have not yet been reported in SSA1 so far. Some secondary endosymbionts were shown to influence fitness and reproduction in SSA species, and in particular within SSA1 (Ghosh et al., 2018; El Hamss et al., 2021). We analyzed the microbiome of SSA1 whiteflies at two levels: (i) endosymbionts, previously defined as intracellular bacteria, and (ii) the meta-microbiome, consisting of all other bacteria, including those unreported or not previously known in Hemipteran insects. Due to their distinct characteristics, these groups were analyzed separately. Unlike endosymbionts, meta-microbiome bacteria can also be transmitted horizontally. This approach ensures a clear distinction, allowing for a more precise analysis of their respective patterns of variation. To investigate microbiome differences associated with whitefly outbreaks, we compared the microbiome profiles of adult *B. tabaci* SSA1 collected from outbreaking and non-outbreaking cassava regions in Uganda. Deep-sequencing techniques provided insights into microbiome variations and their potential relationship with whitefly outbreaks.

The central research question was whether the outbreaking status of cassava whiteflies is associated with differences in their microbiome. To minimize environmental variability, all samples were collected from cassava fields located within a 5 km radius near Lake Victoria, ensuring relatively similar agro-ecological conditions across sites. Sampling focused on a single host plant (cassava), and sites were categorized based on whitefly abundance: outbreaking sites had more than 100 whiteflies per top five leaves, while non-outbreaking sites had substantially fewer individuals. This design allowed us to explore potential microbiome associations with outbreak status under comparable environmental settings.

## Materials and methods

### Whitefly sampling and DNA extraction

In February 2017, whitefly samples were collected from seven districts (Mityana, Mpigi, Wakiso, Kalungu, Masaka, Rakai, and Gomba), with GPS coordinates documented (Ally et al., 2019; El Hamss et al., 2022). A careful sampling strategy, consistent with Ally et al. (2019), was employed, ensuring that the selected samples represented both outbreaking and non-outbreaking populations from the same species and mitotypes. To minimize potential environmental confounding factors, the sampling strategy was designed to target a restricted geographic area in Uganda. All whitefly samples were collected from cassava fields located within a 5 km radius near Lake Victoria. This area is characterized by relatively homogeneous agro-ecological conditions, including uniform crop type (cassava), comparable farming practices, and similar vegetation cover. Both outbreaking and non-outbreaking sites were selected within this confined zone to ensure that microbiome comparisons were conducted under relatively similar environmental conditions.

Specifically, 56 SSA1 adults from cassava plants were collected and preserved in ethanol for subsequent molecular analysis. The outbreak threshold was defined as an abundance of whiteflies exceeding 100 adults on the top five leaves of cassava plants in Ugandan farmers’ fields. Among these, 24 were non-outbreaking, and 32 were outbreaking adult whiteflies (Table 1). For each whitefly individual, DNA was shared between two analyses: part was used for species identification and genetic diversity, while the remainder was used for the present study.

**Table 1:** Sample information and recovered reads in each variable, with A (*Arsenophonus*), E (Endosymbionts), H (*Hemipteriphilus*), Hp (Total Haplotypes), NE (Non-endosymbionts), P (*Portiera*), R (*Rickettsia*), Rp (Replication per treatment), and W (*Wolbachia*) representing average reads per replication.

Genotype	Site	OS	Location x	Location y	Rp	Hp	NE	E	A	W	R	P	H
SSA1-SG1	f1	O	N00.43564	E032.04041	1	2	836	16,206	16,206	0	0	683,726	0
SSA1-SG1	f10	NO	S00.89538	E031.44637	4	2	43,172	3,251	3,163	88	0	805,332	0
SSA1-SG1	f11	O	S00.98063	E031.41873	4	3	160,246	7,059	7,054	5	0	676,064	0
SSA1-SG1	f12	O	S00.12179	E031.75773	8	5	62,574	11,574	6,605	4,969	0	616,831	0
SSA1-SG1	f13	O	N00.17379	E031.92822	4	7	21,882	27,587	6,706	20,882	0	534,922	0
SSA1-SG1	f5	NO	S00.16989	E031.83412	5	2	14,714	2,789	2,710	80	0	655,823	0
SSA1-SG1	f6	NO	S00.33294	E031.70984	4	3	25,309	4,233	3,996	158	0	682,220	78
SSA1-SG1	f8	NO	S00.66515	E031.53927	3	2	13,631	1871	1871	0	0	1,018,733	0
SSA1-SG2	f1	O	N00.43564	E032.04041	4	14	8,583	166,453	35,017	131,437	0	893,487	0
SSA1-SG2	f10	NO	S00.89538	E031.44637	4	15	61,567	130,580	36,561	70,467	23,553	946,639	0
SSA1-SG2	f11	O	S00.98063	E031.41873	5	13	2,499	104,674	23,885	80,789	0	657,978	0
SSA1-SG2	f12	O	S00.12179	E031.75773	2	9	1,355	10,025	1,567	8,458	0	280,633	0
SSA1-SG2	f13	O	N00.17379	E031.92822	4	8	4,813	75,785	9,335	66,450	0	447,448	0
SSA1-SG2	f6	NO	S00.33294	E031.70984	3	12	20,824	71,285	11,018	58,326	1941	430,001	0
SSA1-SG2	f8	NO	S00.66515	E031.53927	1	2	8,959	1756	1756	0	0	584,386	0

DNA extractions of field specimens were obtained from a non-destructive method by overnight incubation of whiteflies in the buffer. Given the haplodiploid nature of the species, only female individuals were selected for analysis. DNA was extracted using a slightly modified protocol based on Delatte et al. (2011). Each whitefly was placed in 50 μL of extraction buffer containing 50 mM KCl, 10 mM Tris-base (pH 8.0), 0.45% IGEPAL CA-630, 0.45% Tween 20, and 500 μg/mL proteinase K (Sigma) in a 96-well PCR plate. Samples were incubated at 65°C for 20 h in a PCR thermocycler, followed by a final heat inactivation at 95°C for 10 min. The resulting DNA extracts were stored at −20°C until further analysis (Delatte et al., 2011).

The identity of whitefly *B. tabaci* species was revealed by both mitochondrial (mtCOI) and nuclear genes (microsatellite markers) (Ally et al., 2019; El Hamss et al., 2022). By carefully selecting samples based on genetic diversity and including both outbreak and non-outbreak populations from the same species and mitotypes, we aimed to conduct a robust and focused analysis, while avoiding redundancy with previous protocol descriptions.

### Library preparation and deep sequencing

The Hi-Seq Illumina platform was used for sequencing the V4-V5 region of the 16S rDNA gene, employing paired-end short reads (average size: 400 bp) to evaluate species diversity and investigate bacterial associations. To mitigate PCR selection bias toward *Portiera*, two PCR amplifications were performed per sample under specific conditions as previously described (El Hamss et al., 2022). Sequence analyses and quality controls were done as previously described (El Hamss et al., 2022).

### Statistical analysis

*Portiera* was excluded from all statistical analyses due to its extremely high prevalence across samples, which significantly skewed the results. To mitigate this, we applied a filtering step in which *Portiera* reads were removed prior to conducting diversity analyses and multivariate statistical tests. This allowed for a more accurate assessment of patterns driven by less dominant bacterial taxa. Welch’s t-test, performed on centered log-ratio (CLR)-transformed data using the ALDEx2 function in R (Gloor, 2015), was employed to assess the differential abundance of microbial genera, as well as to analyze endosymbiont haplotypes and ASVs. Also, the Analysis of the composition of microbiomes (ANCOM) (Mandal et al., 2015) was used to further support the results. To support the identification of differentially abundant taxa, we employed both ANCOM and ALDEx2. However, these methods were applied selectively at specific taxonomic levels based on their respective statistical constraints. ALDEx2 was used only at the endosymbiont level because the ASV-level dataset contained 218 features, which exceeds the optimal feature range for this method and may compromise its performance. In contrast, ANCOM is more robust with larger feature sets and was therefore applied at the ASV level. This selective application ensured appropriate statistical power and reliability of the results. Both analyses were aimed at identifying taxa associated with outbreak status and whitefly mitotypes based on taxon frequency. Additionally, we tested whether endosymbionts coexisted in sympatry and vary according to the site. However, due to insufficient sample sizes per site, we categorized the samples based on GPS coordinates into two main groups to assess the site effect (Supplementary Figure S1). Vegan Library in R (v9) was used to explore the effects of outbreak status on microbial community composition. Distance-based redundancy analysis (Db-RDA) quantified the variance attributed to site, outbreak status, and whitefly mitotypes using the capscale function (Oksanen, 2014). Bray-Curtis distances were subjected to a permutation analysis of variance (PERMANOVA) using the Adonis function of the VEGAN R package. We conducted nonmetric multidimensional scaling (NMDS) and Principal Coordinate Analysis (PCoA) separately for endosymbionts and meta-microbiomes to investigate patterns of separation among microbiomes in different whitefly mitotypes. Outbreak status was tested on endosymbionts and ‘meta-microbiome’ separately due to its non-maternal transmission (Mugerwa et al., 2021b). Shannon, Simpson index, Chao1 and observed ASVs were employed to measure bacterial diversity for each category, with ANOVA used to determine differences between sites and outbreak status. Venn diagrams were used to visualize group-specific cores (El Hamss et al., 2024). Co-occurrence networks illustrated taxa interactions based on frequency of occurrence (presence/absence). The null model analysis was performed in “Vegan” using the ‘quasiswap’ null model method with 999 simulations to assess the significance of the observed co-occurrence pattern in each category compared to randomized data, determining whether the observed network exhibited non-random behavior. Network topology parameters were computed in R using the “coocurence” function. Co-occurrence networks were created using the igraph package, version 2.0.3.

Spearman’s correlation analyzed prevalent ASVs in outbreaking vs. non-outbreaking whiteflies of SSA1-SG1 and SG2. Pearson correlation matrix similarity was tested using the Jennrich test (Zaini and Sharif, 2022) to investigate the equality of correlation matrices generated from endosymbionts in SSA1-SG1 versus SSA1-SG2. Additionally, this test was applied to evaluate the correlation matrices at the meta microbiome level within each outbreaking versus non-outbreaking category. In addition, a PERMANOVA analysis based on Bray-Curtis distances was conducted to evaluate the endosymbionts coexisting in sympatry at each site. To ensure the robustness of the results, a bootstrap procedure with 1,000 iterations was performed.

## Results

### Microbiome composition of SSA1 whiteflies

A total of 42,175,690 16srDNA reads were generated from the bacteria, with an average sequence length of 428 bp (see Table 1 and Supplementary Figure S2). These reads were taxonomically assigned into 218 Amplicon Sequence Variants (ASVs). The sequences have been deposited in GenBank (accession numbers from PV770581 to PV770843), distributed among *Portiera,* meta-microbiome (included all detected bacteria, excluding endosymbionts), and other endosymbionts, and their relative frequency was calculated for each whitefly (Supplementary Figures S2, S3).

*Portiera* was the most common taxon across all sample categories, consistently present regardless of investigated factors. In non-outbreaking samples of SSA1-SG1, *Portiera* averaged 795,213 reads (± 460,131), accounting for 0.89 (± 0.26) of the relative frequency (Table 1). Similar patterns were observed in other sample groups*. Portiera*, due to its high prevalence, was then excluded from all statistical analyses to prevent skewing the results (Supplementary Figure S2).

**Figure 1:**
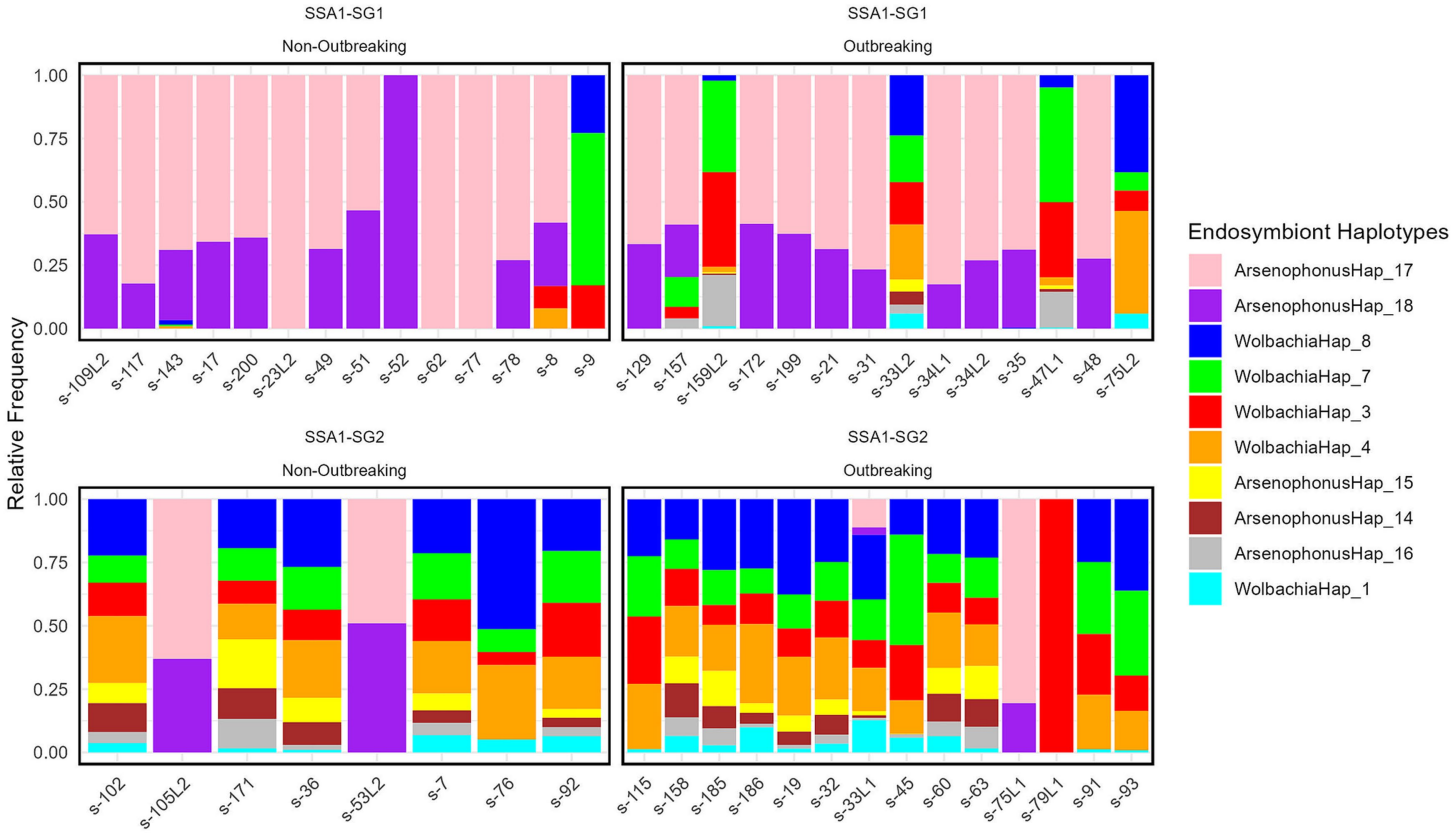
Relative frequencies of endosymbionts, excluding *Portiera*, among the top 10 abundant haplotypes in two SSA1 mitotypes (SSA1-SG1 and SSA1-SG2) and two outbreak statuses. “Outbreaking” refers to sites where whiteflies were collected and exceeded 100 individuals per top 5 cassava leaves. All top taxa were significantly different based on Welch’s t-test performed on centered log-ratio (CLR)-transformed data using the ALDEx2 function in R, except for *ArsenophonusHap*_16. While the same test on outbreaking in each whitefly mitotype was not significantly different.

The distribution of the meta-microbiome and endosymbionts across all samples is illustrated in Supplementary Figure S3. Endosymbionts were analyzed separately at the whitefly mitotype level (Figure 1). *Portiera* frequency in SSA1-SG1 was lower compared to SSA1-SG2, irrespective of outbreaking status. Endosymbiont relative frequency varied significantly across mitotypes (Supplementary Table S1) but not with outbreaking status (Supplementary Table S2) or site (Supplementary Table S3). ALDEX X 2 and ANCOM analyses identified *Arsenophonus* Hap_17 and Hap_18 as the most abundant in SSA1-SG1, with a significantly lower presence in SSA1-SG2 (Figure 1; Supplementary Table S1).

**Figure 2:**
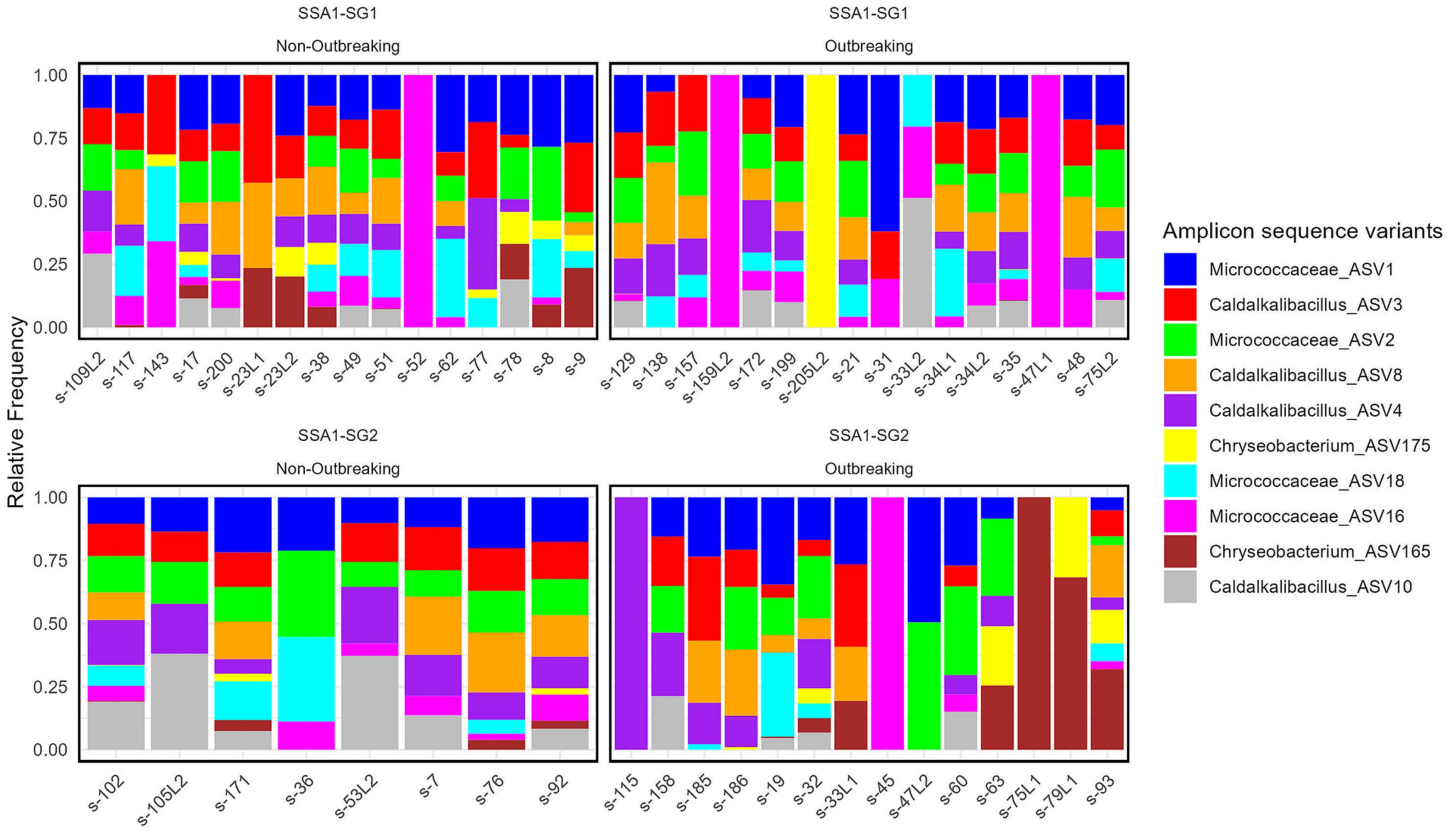
Relative frequencies of meta microbiome among top 10 abundant ASVs in two SSA1 mitotypes (SSA1-SG1 and SSA1-SG2) and two outbreaking statuses. ‘Outbreaking’ denotes sites where whiteflies were collected and exceeded 100 per top 5 cassava leaves.

Meta-microbiome prevalence was highest in SSA1-SG1, particularly in outbreaking samples, whereas SSA1-SG2 showed a significantly lower frequency (Table 1; Figure 2). Among the 218 ASVs, 77 varied significantly across categories, suggesting their potential role in whitefly population dynamics (Table 1; Figure 2).

### Outbreak dynamics and microbiome composition in SSA1 whiteflies

**Figure 3:**
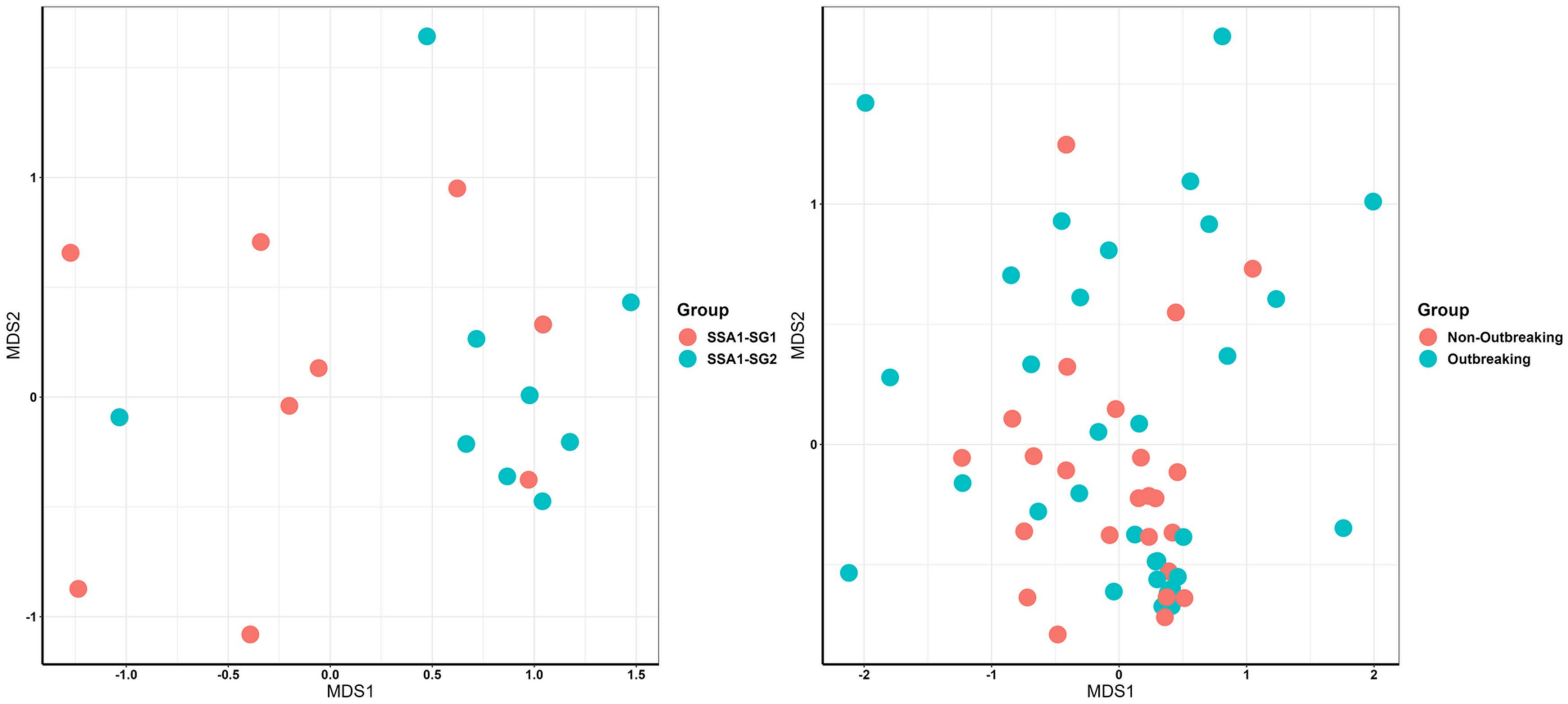
NMDS representation of endosymbiont composition (left) in two whitefly SSA1 mitotypes (SSA1-SG1 and SSA1-SG2) and meta-microbiome composition (right) in two outbreaking statuses. Each dot represents the dimension of each endosymbiont or meta-microbiome in the MDS1 and MDS2 scales.

For endosymbionts, the NMDS stress was 0.04 with a *p*-value of 0.001, as determined by ADONIS and Capscale using Bray–Curtis metrics (Figure 3) and further supported by PcoA (Supplementary Figure S6). PCoA plots revealed partial separation between outbreaking and non-outbreaking groups, suggesting differences in community composition. Outbreaking status was not shown here as it did not differ between both mitotypes. The results revealed that endosymbionts from SSA1-SG1 clustered separately from SSA1-SG2 and exhibited a significant difference in endosymbiont composition (*p* < 0.01), determined by Adonis and capscale (Figure 3).

The meta-microbiome composition of whitefly mitotypes in different outbreak locations also differed significantly based on the two complementary tests (Figure 3; NMDS stress = 0.18, *p*-value = 0.001). PCoA plots revealed partial separation between outbreaking and non-outbreaking groups, suggesting differences in community composition (Supplementary Figure S7).

**Figure 4:**
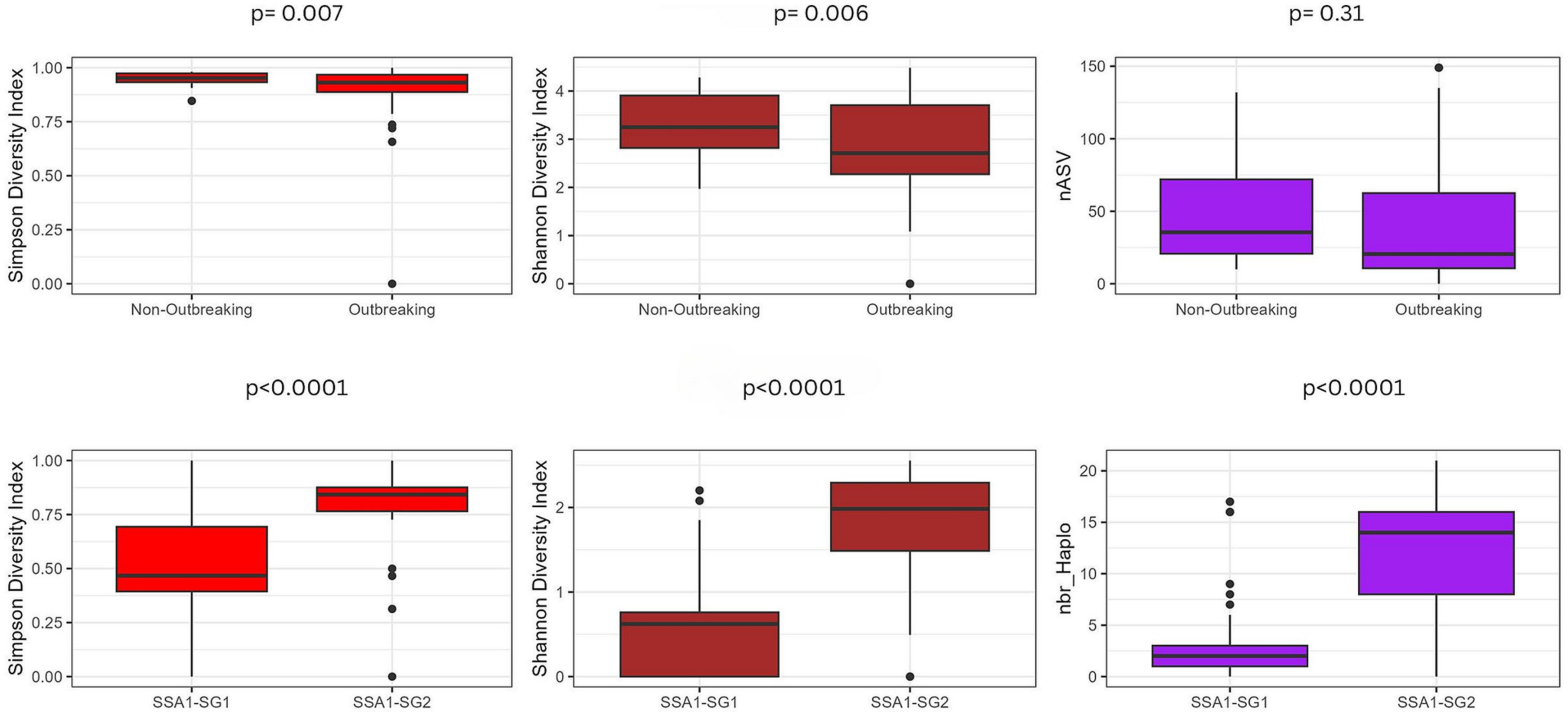
Diversity metrics measured by Shannon and Simpson, along with the number of haplotypes in endosymbionts in SSA1-SG1 and SSA1-SG2 and number of ASVs in meta-microbiome (upper) across two outbreaking statuses.

Simpson, Shannon and Chao1 indices showed significant endosymbiont diversity between SSA1-SG1 and SG2 (Figure 4, *p* < 0.0001). SSA1-SG2 displayed higher species diversity than SSA1-SG1 (Figure 4). However, at the meta-microbiome level, no differences in diversity indices between SSA1-SG1 and SSA1-SG2 were observed. Instead, their outbreaking status was significantly different in Simpson (*p* = 0.007) and Shannon (*p* = 0.006) indices (Figure 4). Chao1 indice further supported that (*p* = 0.040) The number of ASVs was not significant. Non-outbreaking whiteflies showed the highest Shannon and Simpson indices compared to outbreaking ones (Figure 4).

### Core endosymbionts analysis by whitefly mitotype

**Figure 5:**
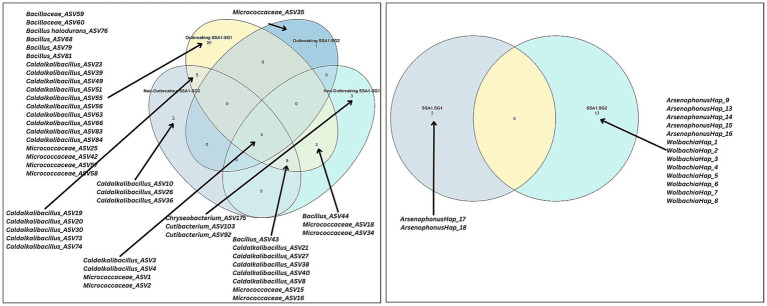
Venn diagram illustrating shared and unique cores across mitotypes at the endosymbiont level and across outbreaking statuses and mitotypes at the meta microbiome level. The Venn diagram was constructed based on two criteria: a detection level of 0.001 (present in at least 0.1% of samples) and a prevalence cutoff of 0.5 (present in at least 50% of samples). Arrows indicate the number of cores in each category, as listed in the graph.

All tested whiteflies collectively yielded a total of 15 haplotypes belonging to endosymbionts. None of these were shared between SSA1-SG1 and SSA1-SG2 (Figure 5). Thirteen unique haplotypes were identified in SSA1-SG2, whereas only two were observed in SSA1-SG1.

### Core meta-microbiome analysis by outbreaking status and mitotype

The meta-microbiome showed greater diversity, with 218 ASVs detected across all whitefly samples. (Figure 5). The number of ASVs in the outbreaking SSA1-SG1 was significantly larger than the non-outbreaking whiteflies while the smallest number of ASVs (1) was observed in the outbreaking SSA1-SG2. Additionally, the outbreaking SSA1-SG1 contained the largest number of outbreaking SSA1-SG1 ASVs, reaching 20, followed by non-outbreaking SSA1-SG1 and non-outbreaking SSA1-SG2, each harboring 3 unique ASVs. A total of 20 ASVs appeared to be shared in at least two groups.

### Co-occurrence network and correlation analysis of endosymbionts

The co-occurrence network analysis revealed non-random interactions among taxa in SSA1-SG1 and SSA1-SG2 whitefly mitotype, supported by null model tests (*p*-values: 0.01 and 0.001, respectively). The SSA1-SG2 network displayed higher complexity, with 18 nodes and 134 edges (average connectivity of 14.89), compared to SSA1-SG1 which displayed 15 nodes and 26 edges (average connectivity of 3.46). SSA1-SG2 also exhibited a more clustered topology (clustering coefficient: 0.92 vs. 0.34) and lower modularity (0.034 vs. 0.24) (Supplementary Figure S4).

**Figure 6:**
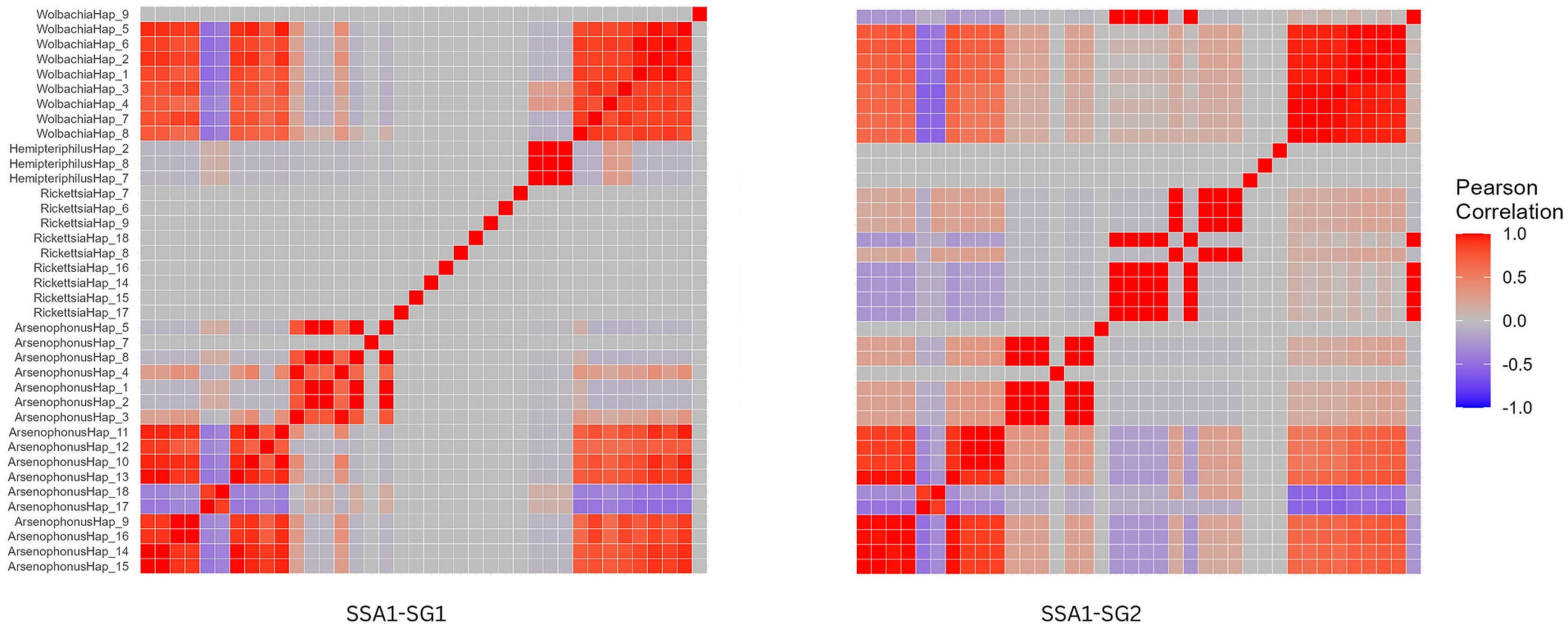
Heatmap of pairwise Pearson correlation coefficients for the correlations among endosymbionts within each whitefly mitotype (SSA1-SG1 and SSA1-SG2). A high absolute value of the Pearson correlation coefficient corresponds to a high degree of correlation. Dark red indicates a high positive correlation, while dark blue indicates a high negative correlation. Light red and light blue indicate low degrees of positive and negative correlation, respectively, and gray indicates no correlation.

Correlation heatmaps showed strong positive associations among *Arsenophonus* haplotypes 1–3 and 9–16, and *Wolbachia* haplotypes 1–9 in both mitotypes. However, *Hemipteriphilus* haplotypes 2, 7, and 8 were positively correlated only in SSA1-SG1, while *Rickettsia* haplotypes 6, 7, and 9 were positively correlated only in SSA1-SG2 (Figure 6).

### Co-occurrence network and correlation analysis of meta-microbiome

**Figure 7:**
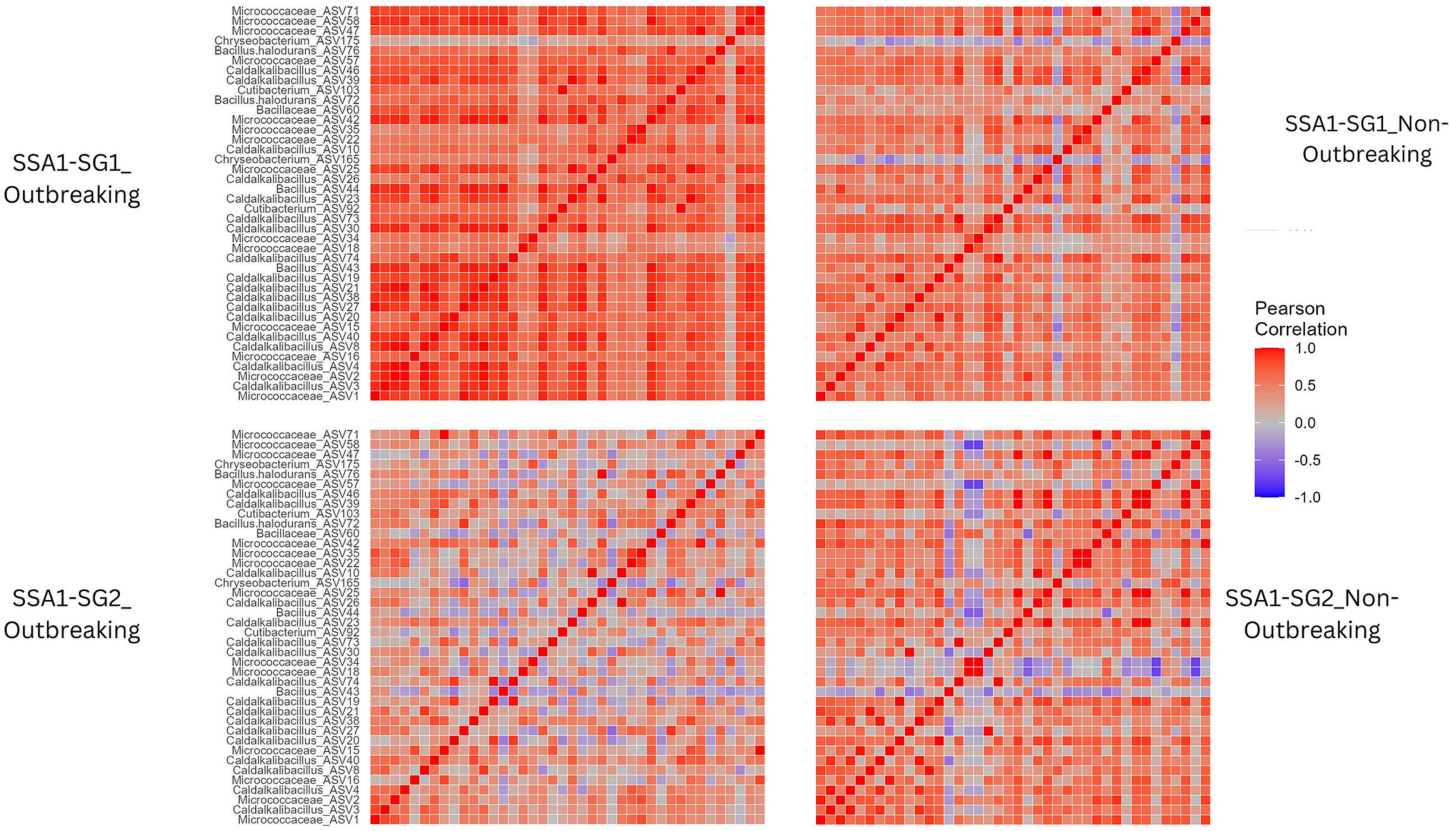
Heatmap of pairwise Pearson correlation coefficients for the correlations among meta-microbiome within each outbreaking status and mitotype. A high absolute value of the Pearson correlation coefficient signifies a strong correlation. Dark red represents a strong positive correlation, while dark blue signifies a strong negative correlation. Light red and light blue indicate weaker positive and negative correlations, respectively, and gray denotes no correlation.

Meta-microbiome co-occurrence networks revealed distinct patterns of microbe-microbe interactions influenced by outbreak dynamics. In SSA1-SG1 outbreaking regions, the network displayed a non-random structure (*p* = 0.001), highlighting the impact of outbreaks on community composition. Similar structured assembly processes were observed in other networks, including SSA1-SG1_non-outbreaking, SG2_outbreaking, and SSA1-SG2_non-outbreaking (*p* = 0.001). Compared to endosymbionts, the meta-microbiome networks were more complex due to higher microbial diversity and influenced by both whitefly mitotype and outbreak status (Table 2; Supplementary Figure S5). The strongest positive correlations were observed in SSA1-SG1 from outbreaking regions, emphasizing the intricate relationship between the meta- microbiome and whitefly outbreaks (Figure 7).

**Table 2:** Topological parameters of the co-occurrence networks in the different whitefly groups within SSA1 species and in relation to their outbreaking statuses.

Topology parameters	SSA1-SG1 Outbreaking	SSA1-SG1Non Outbreaking	SSA1-SG2 Outbreaking	SSA1-SG2 Non Outbreaking
Average path length	1.7	2.5	2.9	1
Diameter	4	6	6	1
Clustering coefficient	0.8	0.5	0.4	1
Modularity index	0.1	0.3	0.6	0.8
Total nodes	133	108	36	35
Total edges	3,620	783	61	41
Average degree	54.4	14.5	3.4	2.3
Network coverage	50.8	41	13	13

## Discussion

In this study, we investigated the microbiome composition and diversity between outbreaking and non-outbreaking SSA1 *B. tabaci* populations on cassava in Uganda. Significant variations were demonstrated between endosymbionts and meta-microbiome composition, diversity, and structure between the outbreaking and non-outbreaking sites of SSA1-SG1 and SSA1-SG2 mitotypes.

Endosymbiont diversity was not influenced by outbreaking status, whereas the meta-microbiome was structured by outbreaking status. A specific 20 core ASVs were unique to the outbreaking SSA1-SG1, including different strains of *Caldalkalibacillus*, and species belonging to *Bacillaceae*, and *Micrococcaceae*. Although SSA1-SG1 and SG2 are the same species (Ally et al., 2019; Mugerwa et al., 2021b; Ally et al., 2023; Campbell et al., 2023), we found that their microbiome networks, diversity, and composition differed significantly. It is not uncommon to observe variations within populations, where one population may be more ‘aggressive’ or ‘fit’ than the other, as seen in studies with fruit flies or other insects such as the harlequin ladybird *Harmonia axyridis* (Vandereycken et al., 2012). These variations within populations can trigger invasive behavior, and they are often linked to ecological and evolutionary factors that influence fitness and adaptability. This highlights the importance of considering population-level differences when studying invasion dynamics, moving beyond taxonomic classifications to understand the biological drivers of outbreaks.

We examined two levels of microbial diversity: endosymbionts and meta-microbiome. Endosymbionts, primarily *Portiera*, *Arsenophonus*, and *Wolbachia*, showed no variation by site or outbreak status, aligning with mitochondrial profiles. *Candidatus hemipteriphilus* was found in 1.7% of samples, and *Rickettsia* in 3.5%, with 9 haplotypes identified. These findings are consistent with previous studies demonsarting that endosymbiont associations tend to be conserved, not only at the mitotype level but also across different whitefly species beyond Uganda (Kanakala and Ghanim, 2019). A large-scale study of over 2000 whiteflies revealed a non-random distribution of facultative endosymbionts among distinct *B. tabaci* species (Zchori-Fein et al., 2014). *Arsenophonus* was primarily found in the Mediterranean (MED) species complex and Middle East-Asia Minor 2 (MEAM2), whereas *Wolbachia* was absent in MEAM1 individuals (Zchori-Fein et al., 2014; Wang et al., 2019).

In our study, the contrasting patterns observed between endosymbionts and the environmentally acquired meta-microbiome likely reflect differences in their modes of transmission and ecological roles. The endosymbionts, which are maternally inherited, showed relatively stable distributions across both outbreaking and non-outbreaking populations, suggesting they are conserved components of the whitefly’s internal microbiota. This stability implies a foundational role in host biology, potentially linked to essential functions such as nutrient provision or reproductive manipulation.

Beyond endosymbionts, we found significant differences in the diversity, composition, and network properties of the meta-microbiome between SSA1-SG1 and SG2, indicating that microbiome variability exists even within the same species. Genetic makeup and outbreak status likely influence this variation. Given that these bacteria are environmentally acquired, their composition is likely shaped by local factors such as cassava phyllosphere communities, climatic conditions, or insect density. This variability may reflect a more flexible, short-term form of microbial association, potentially enabling whiteflies to respond rapidly to environmental stressors or host plant defenses during outbreaks. The clearer structuring of the meta-microbiome by outbreak status, compared to endosymbionts, supports the idea that environmentally acquired bacteria might contribute to outbreak dynamics by influencing host adaptability or competitive success under high-density conditions.

To better understand the role of these different strains, deeper sequencing with longer fragments is needed.

This study highlights distinct meta-microbiome structures in SSA1 whiteflies from outbreak sites, emphasizing the role of specific bacterial groups in whitefly performance. Twenty ASV groups, including *Bacillus, Caldalkalibacillus*, and *Micrococcaceae*, were predominantly found in SSA1-SG1 individuals from outbreak sites, suggesting their potential involvement in local adaptation and/or increased fitness. *Caldalkalibacillus* (ASV3 and ASV4) and *Micrococcaceae* (ASV1 and ASV2) were also abundant and cosmopolitan across all tested categories, suggesting that these taxa might not be directly influenced by outbreak status or linked to a specific mitotype. Their roles in their host biology should be further investigated. The gut microbiome are known to play a key role in helping whiteflies to adapt to different plants, potentially enhancing their performance in sites with favorable environmental conditions (Su et al., 2016; Santos-Garcia et al., 2020). For instance, an increase in *Mycobacterium* bacteria in whiteflies feeding on pepper helped them to break down harmful substances, improving their survival (Santos-Garcia et al., 2020). Similarly, in aphids transferred to different *Solanaceae* plants, a decrease in *Buchnera* symbionts and an increase in *Pseudomonas* were observed, suggesting that microbiome shifts may be linked to host plant adaptability. However, aphids on pepper showed a smaller increase in *Pseudomonas*, potentially affecting their ability to thrive on this plant (He et al., 2021). It is worth noting that in our study, all whiteflies were collected from different sites but from the same host plant, cassava, within a monoculture agricultural system in Uganda. This reduces the likelihood that the observed microbiome variations were due to host plant adaptation. Instead, the differences may reflectsite-specific environmental factors or intrinsic microbiome community dynamics. Might be influencing the outbreak patterns. In microbial ecology, the complexity of co-occurrence networks is often linked to ecological stability and resilience. In our study, the microbial network in SSA1-SG2 was notably more complex than in SSA1-SG1. This greater connectivity may indicate a more robust microbial structure, potentially buffering the host population against perturbations such as pathogen invasion or environmental stress. Such resilience could contribute to the lower outbreak tendency observed in SSA1-SG2 populations. Further studies are needed to confirm whether this network complexity supports greater niche stability or functional redundancy in the whitefly microbiome.

A related study by Li et al. (2023) on wheat microbiome found that surface-associated (phyllosphere) communities were more complex and stable than internal (endosphere) ones, with key bacterial taxa like *Pantoea*, *Massilia*, and *Pseudomonas* structuring the community. While direct comparisons between plant and insect microbiomes are limited by biological differences, this highlights a broader ecological principle: microbiome complexity can reflect and influence host-environment interactions. Given that whiteflies are sap-feeders, the microbiomes of both the insect and its host plant may interact. Future investigations into the cassava microbiome may reveal how plant-associated microbes influence whitefly microbial communities, potentially shaping outbreak dynamics and ecological adaptation.

This study examined the link between outbreaking whiteflies and their meta-microbiome, uncovering non-random differences in bacterial communities between outbreaking and non-outbreaking sites. Co-occurrence analysis identified ecological factors shaping the microbiome in SSA1. *B. tabaci* likely acquires *Caldalkalibacillus* from environmental sources, which may assist in digestion or stress resistance (de Jong et al., 2024). The presence of 10 *Caldalkalibacillus* strains in outbreak sites suggests a potential role in outbreak dynamics.

While bacteria like Bacillaceae and Pseudomonadaceae are traditionally seen as pathogens for their hosts (Deka et al., 2022), our findings suggest they may have a positive effect in outbreaking populations. However, this effect could also be the opposite—we do not know for certain. It is possible that in sites with high whitefly densities, these bacteria act as pathogens, spreading rapidly within the population due to the close proximity of thousands of individuals during outbreaks. This study provides a snapshot of the whitefly microbiome during a defined outbreak event in Uganda, using a geographically and environmentally constrained sampling strategy to reduce confounding variables. Although sampling was limited to a single country and time point, the observed patterns of microbial diversity and composition. We acknowledge that broader temporal and spatial sampling, as well as functional validation experiments, would help establish causality and generalize findings across regions. Nevertheless, our results offer important insights and generate hypotheses for future research on the ecological role of microbiomes in whitefly outbreaks.

This also raises the question of whether these bacterial taxa could also contribute to the eventual decline of an outbreak. Given that we collected samples at a single time point and from a limited number of insects, we must be cautious in extrapolating our results to the broader population. Additionally, considering that these bacterial genera are known pathogens, multiple scenarios should be explored to fully understand their role in whitefly outbreaks.

Previous studies on multicolored Asian lady beetle (*Harmonia axyridis*) demonstrated that Micrococcaceae may enhance nutrient absorption during oviposition, potentially aiding in the digestion of cell walls and lignocelluloses (Hu et al., 2018; Huang et al., 2022). The Micrococcaceae family is frequently found in the midguts of a wide range of insect species (Uzmi et al., 2019; Hathnagoda et al., 2024). For example, the genus Micrococcus, *Micrococcus luteus* was detected in both the pre-pupal and adult stages of the dissected gut of a subcortical beetle (*Agrilus planipennis*) (Vasanthakumar et al., 2008). In our study, this bacteria was detected at the family level with different strains observed, longer sequences are also needed to identify them at the species level to see which species are present in SSA1 whiteflies. Micrococcaceae can be transferred to whiteflies while feeding on sap leaves or while coming in contact with an infected environment but this should be investigated.

On a broader scale, our results support the observation that differences in outbreak dynamics shape the meta-microbiome of SSA1 whiteflies. Its diversity and composition differed significantly between non-outbreaking and outbreaking populations across all sites. Future studies on the drivers of local invasions or epidemic emergence should consider the microbiome as a potential contributing factor, with a focus on identifying the specific bacteria or bacterial combinations involved.

## Data Availability

The original contributions presented in the study are publicly available. This data can be found here: https://www.ncbi.nlm.nih.gov/genbank/, accession numbers PV770581 to PV770843.

## Supplementary material

SUPPLEMENTARY FIGURE S1 Visualization of the site clusters based on their GPS coordinates. The points represent the sites, colored by their assigned cluster. The red "X" marks are the centroids of the two clusters.

SUPPLEMENTARY FIGURE S2 Relative frequencies of meta-microbiome, endosymbionts, and *Portiera* in two SSA1 groups and two outbreaking statuses. 'Outbreaking' denotes sites where whiteflies were collected and exceeded 100 per top 5 cassava leaves.

SUPPLEMENTARY FIGURE S3 Relative frequencies of meta-microbiome and endosymbionts after filtering out *Portiera* in Two SSA1 groups and two outbreaking statuses. 'Outbreaking' denotes sites where whiteflies were collected and exceeded 100 per top 5 cassava leaves.

SUPPLEMENTARY FIGURE S4 Co-occurrence network of endosymbionts within each whitefly mitotype (left: SSA1-SG1, right: SSA1-SG2). Gray lines represent weaker interactions compared to black lines, indicating stronger interactions. The distance between bubbles reflects the frequency of occurrence: closer bubbles indicate a stronger frequency across tested samples, while more distant bubbles indicate a weaker frequency. Colored bubbles indicate endosymbionts that co-occur together, while gray ones indicate they exist without interaction. Both network patterns were significantly different from those occurring by chance, based on non-random tests (p = 0.011 and 0.001 for SSA1-SG1 and SSA1-SG2, respectively).

SUPPLEMENTARY FIGURE S5 Co-occurrence network of meta-microbiome within each whitefly mitotypes and outbreaking status. Gray lines represent interactions within bacteria. The distance between bubbles reflects the frequency of occurrence: closer bubbles indicate a stronger frequency across tested samples, while more distant bubbles indicate a weaker frequency.

SUPPLEMENTARY FIGURE S6 Principal Coordinate Analysis (PCoA) based on Bray-Curtis distances, showing differences in endosymbiont composition according to whitefly mitotype. Each dot represents the endosymbionts composition of a single whitefly sample. PCoA1 and PCoA2 explain 42.6% and 16.1% of the variation, respectively.

SUPPLEMENTARY FIGURE S7 Principal Coordinate Analysis (PCoA) based on Bray-Curtis distances, showing differences in meta-microbiome composition according to outbreak status. Each dot represents the meta-microbiome composition of a single whitefly sample. PCoA1 and PCoA2 explain 18.5% and 12.6% of the variation, respectively.

SUPPLEMENTARY TABLE S1 Differential abundance and effect of mitotype on endosymbiont taxa using ALDEx2 Centered Log-Ratio: Includes Taxa (e.g., *Arsenophonus*), abundance in experimental conditions (we.ep, we.eBH, wi.ep, wi.eBH), ratio of abundance across conditions (rab.all, rab.win.sg1, rab.win.sg2), differences between mitotypes (diff.btw, diff.win), effect Size (effect, effect.low, effect.high), and overlap.

SUPPLEMENTARY TABLE S2 Differential abundance and effect of outbreaking status on endosymbiont taxa using ALDEx2 Centered Log-Ratio: Includes Taxa (e.g., *Arsenophonus*), abundance in experimental conditions (we.ep, we.eBH, wi.ep, wi.eBH), ratio of abundance across conditions (rab.all, rab.win.sg1, rab.win.sg2), differences between mitotypes (diff.btw, diff.win), effect size (effect, effect.low, effect.high), and overlap."

SUPPLEMENTARY TABLE S3 Differential abundance and affect of site on endosymbiont taxa using ALDEx2 Centered Log-Ratio: Includes taxa (e.g., *Arsenophonus*), abundance in experimental conditions (we.ep, we.eBH, wi.ep, wi.eBH), ratio of abundance across conditions (rab.all, rab.win.sg1, rab.win.sg2), differences between mitotypes (diff.btw, diff.win), effect size (effect, effect.low, effect.high), and overlap."

SUPPLEMENTARY TABLE S4 Species annotation (ASV ID, genus/species name) and statistical results of significant differences identified by the ANCOM test, analyzed in relation to outbreaking status and mitotype.

SUPPLEMENTARY TABLE S5 List of core microbiome detected in each tested whitefly by outbreaking status and mitotype.
