# Supplementary figures and images for: Outbreak-driven differences in the microbiome composition and diversity of two cassava whitefly Bemisia tabaci mitotypes SSA1-SG1 and SSA1-SG2
Source: Front Microbiol. 2025 Jul 11;16:1597836. doi: 10.3389/fmicb.2025.1597836 (PMC12289618; doi:10.3389/fmicb.2025.1597836)

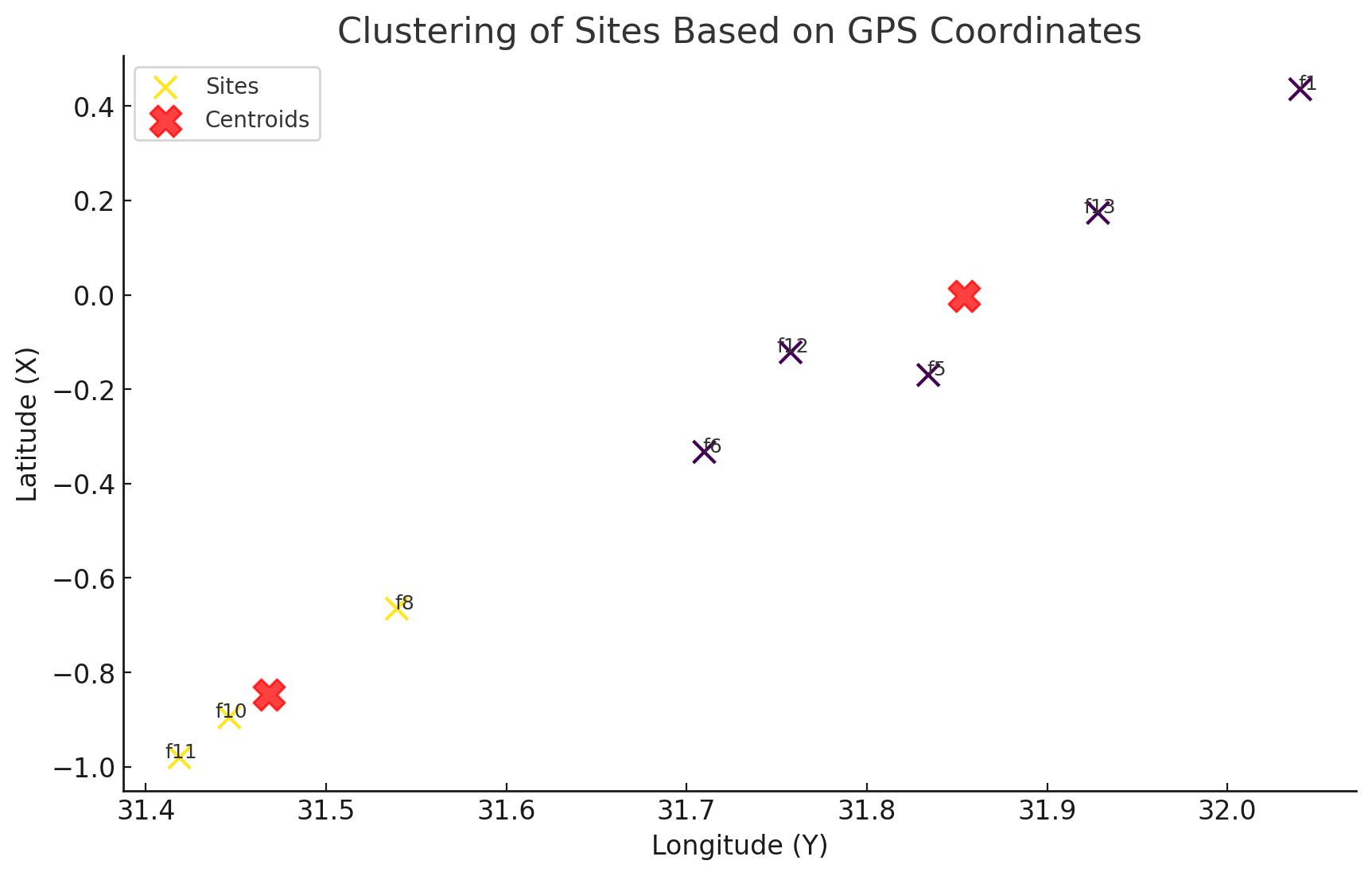

Supplement: SUPPLEMENTARY FIGURE S1 — Visualization of the site clusters based on their GPS coordinates. The points represent the sites, colored by their assigned cluster. The red "X" marks are the centroids of the two clusters. [file Image_1.png]

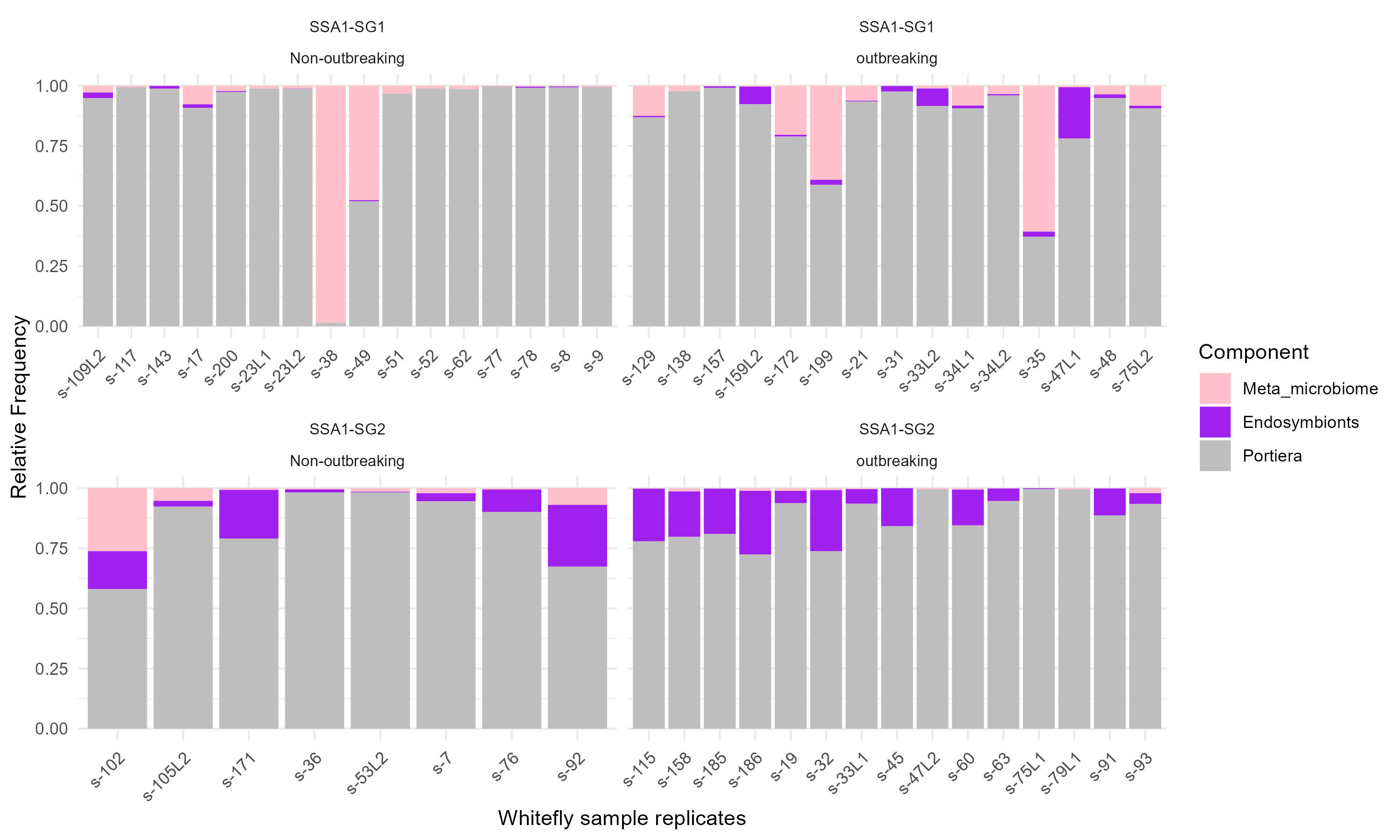

Supplement: SUPPLEMENTARY FIGURE S2 — Relative frequencies of meta-microbiome, endosymbionts, and Portiera in two SSA1 groups and two outbreaking statuses. 'Outbreaking' denotes sites where whiteflies were collected and exceeded 100 per top 5 cassava leaves. [file Image_2.jpeg]

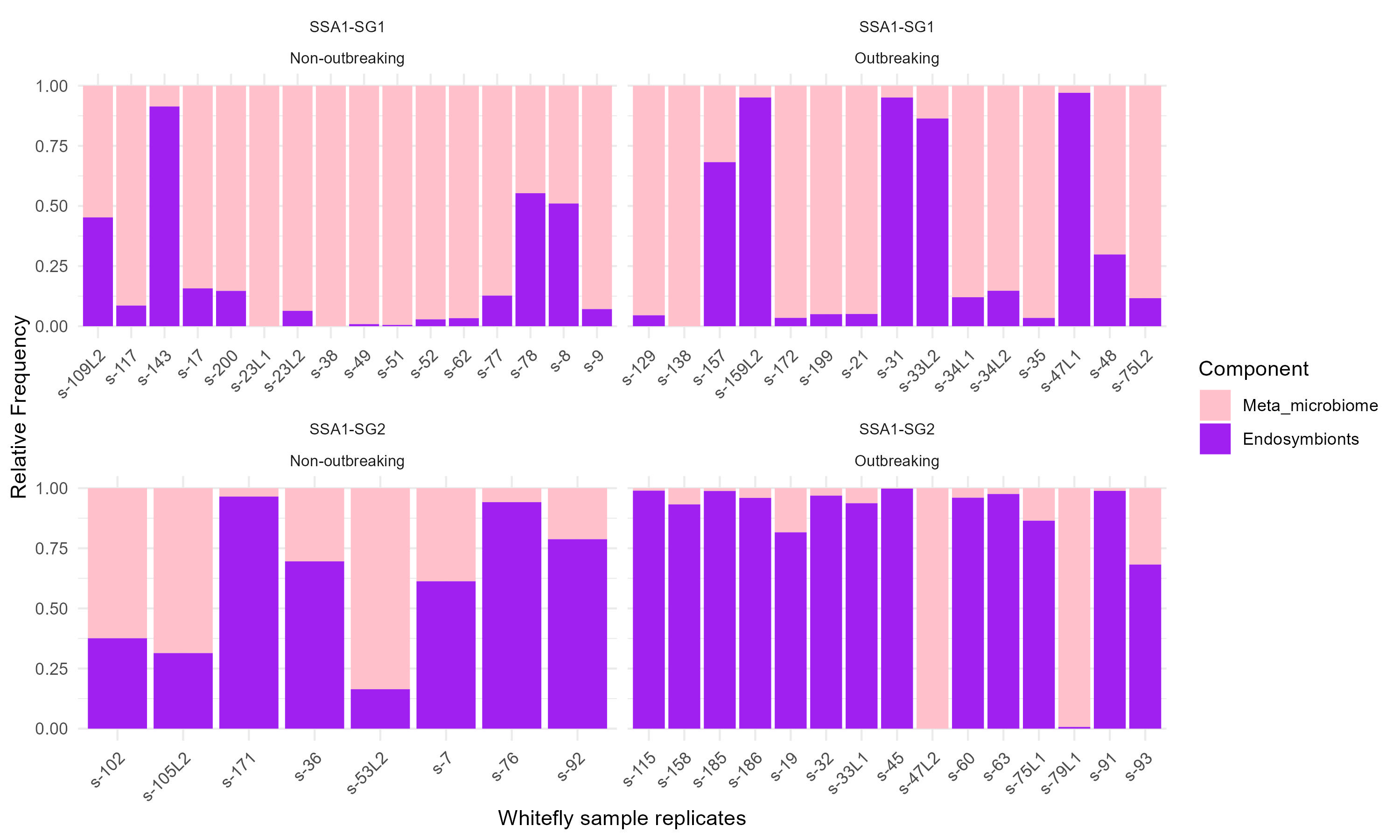

Supplement: SUPPLEMENTARY FIGURE S3 — Relative frequencies of meta-microbiome and endosymbionts after filtering out Portiera in Two SSA1 groups and two outbreaking statuses. 'Outbreaking' denotes sites where whiteflies were collected and exceeded 100 per top 5 cassava leaves. [file Image_3.jpeg]

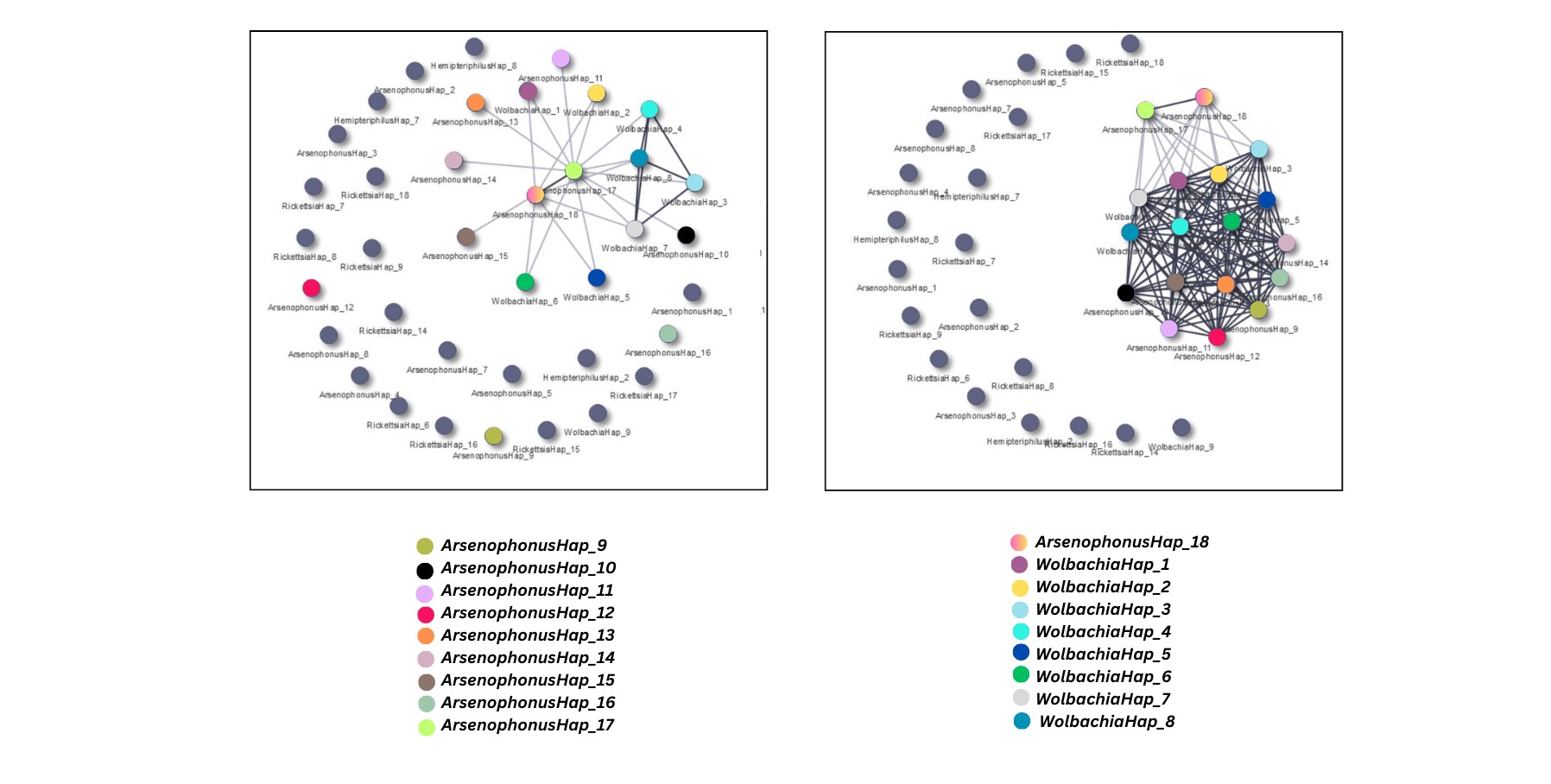

Supplement: SUPPLEMENTARY FIGURE S4 — Co-occurrence network of endosymbionts within each whitefly mitotype (left: SSA1-SG1, right: SSA1-SG2). Gray lines represent weaker interactions compared to black lines, indicating stronger interactions. The distance between bubbles reflects the frequency of occurrence: closer bubbles indicate a stronger frequency across tested samples, while more distant bubbles indicate a weaker frequency. Colored bubbles indicate endosymbionts that co-occur together, while gray ones indicate they exist without interaction. Both network patterns were significantly different from those occurring by chance, based on non-random tests (p = 0.011 and 0.001 for SSA1-SG1 and SSA1-SG2, respectively). [file Image_4.jpeg]

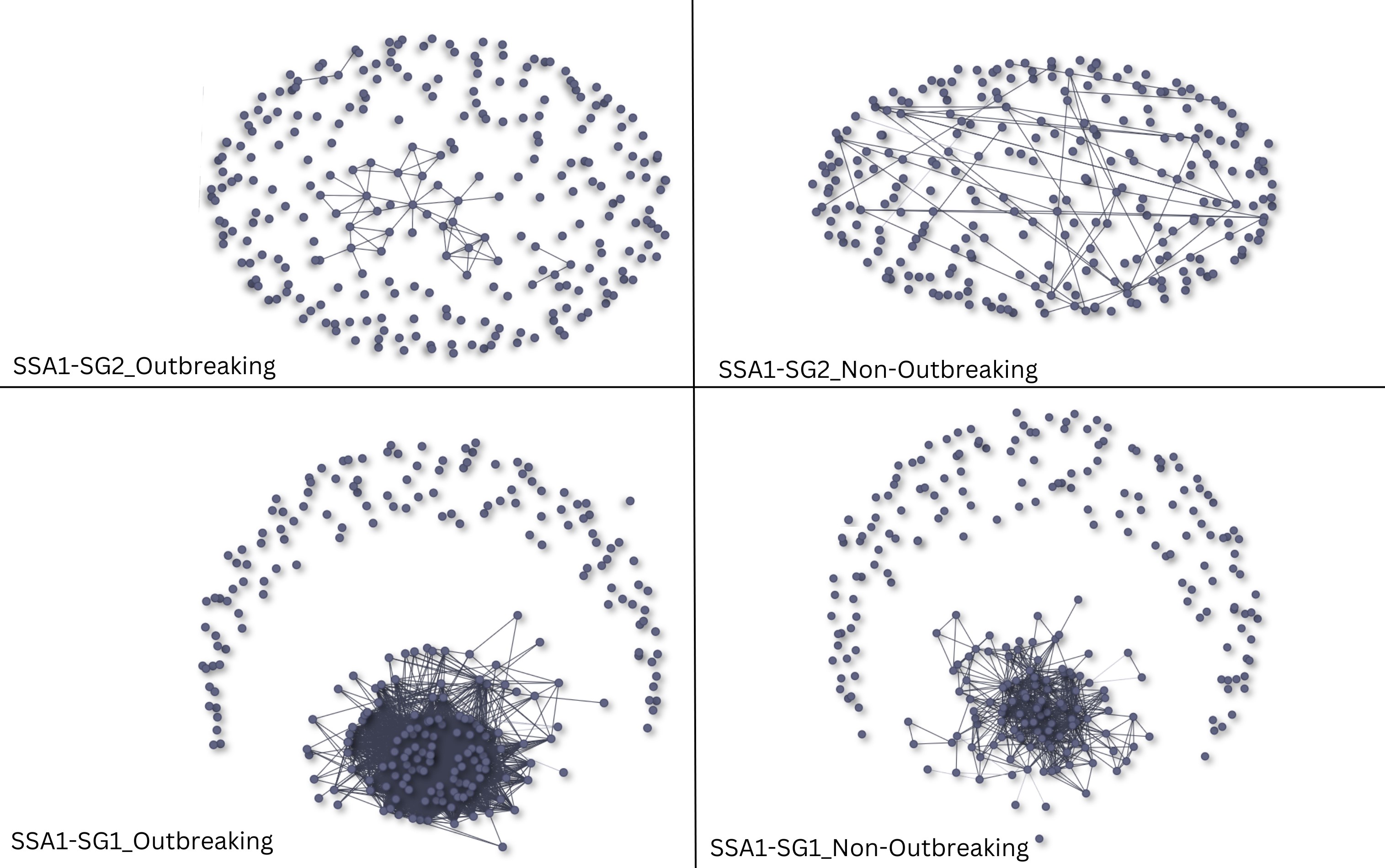

Supplement: SUPPLEMENTARY FIGURE S5 — Co-occurrence network of meta-microbiome within each whitefly mitotypes and outbreaking status. Gray lines represent interactions within bacteria. The distance between bubbles reflects the frequency of occurrence: closer bubbles indicate a stronger frequency across tested samples, while more distant bubbles indicate a weaker frequency. [file Image_5.jpeg]

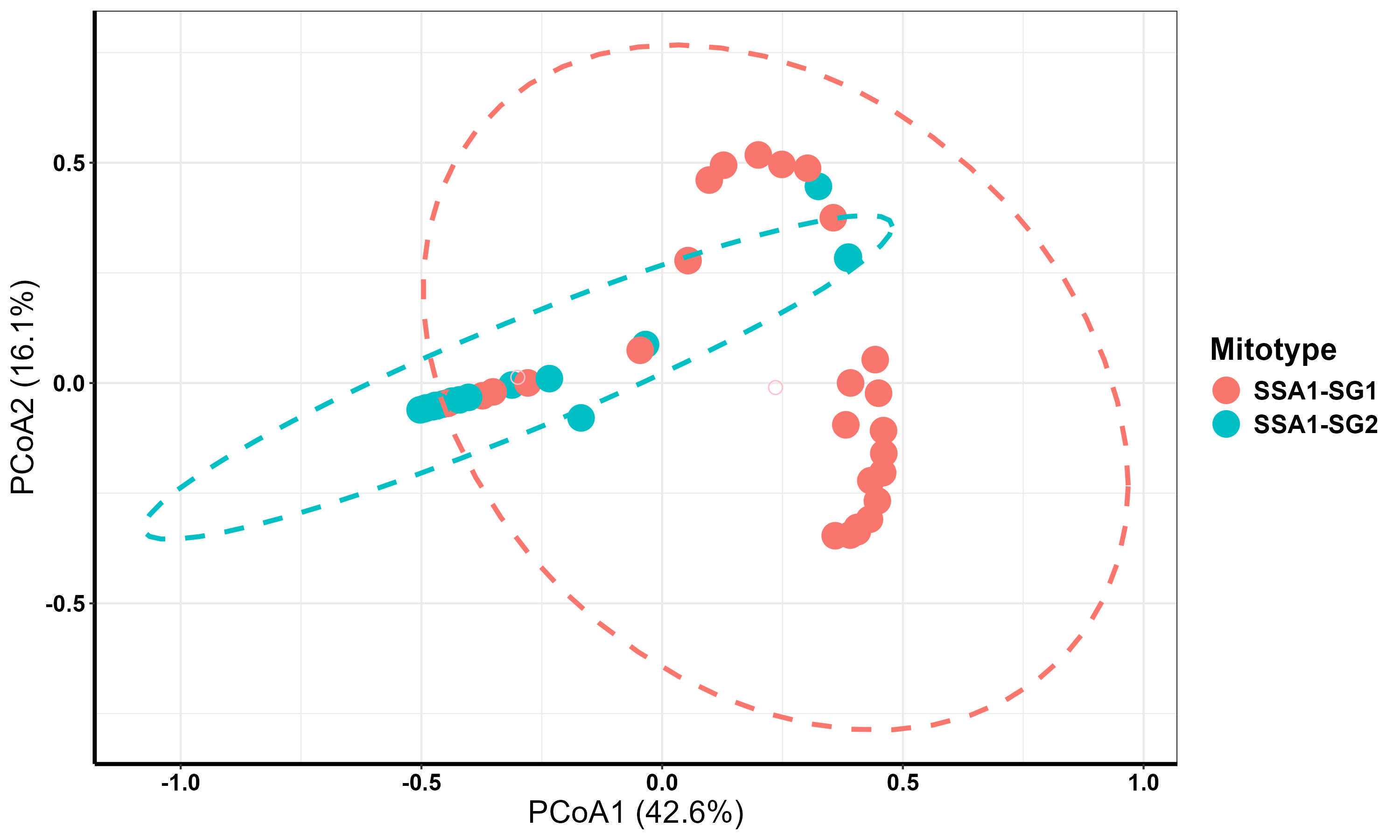

Supplement: SUPPLEMENTARY FIGURE S6 — Principal Coordinate Analysis (PCoA) based on Bray-Curtis distances, showing differences in endosymbiont composition according to whitefly mitotype. Each dot represents the endosymbionts composition of a single whitefly sample. PCoA1 and PCoA2 explain 42.6% and 16.1% of the variation, respectively. [file Image_6.jpeg]

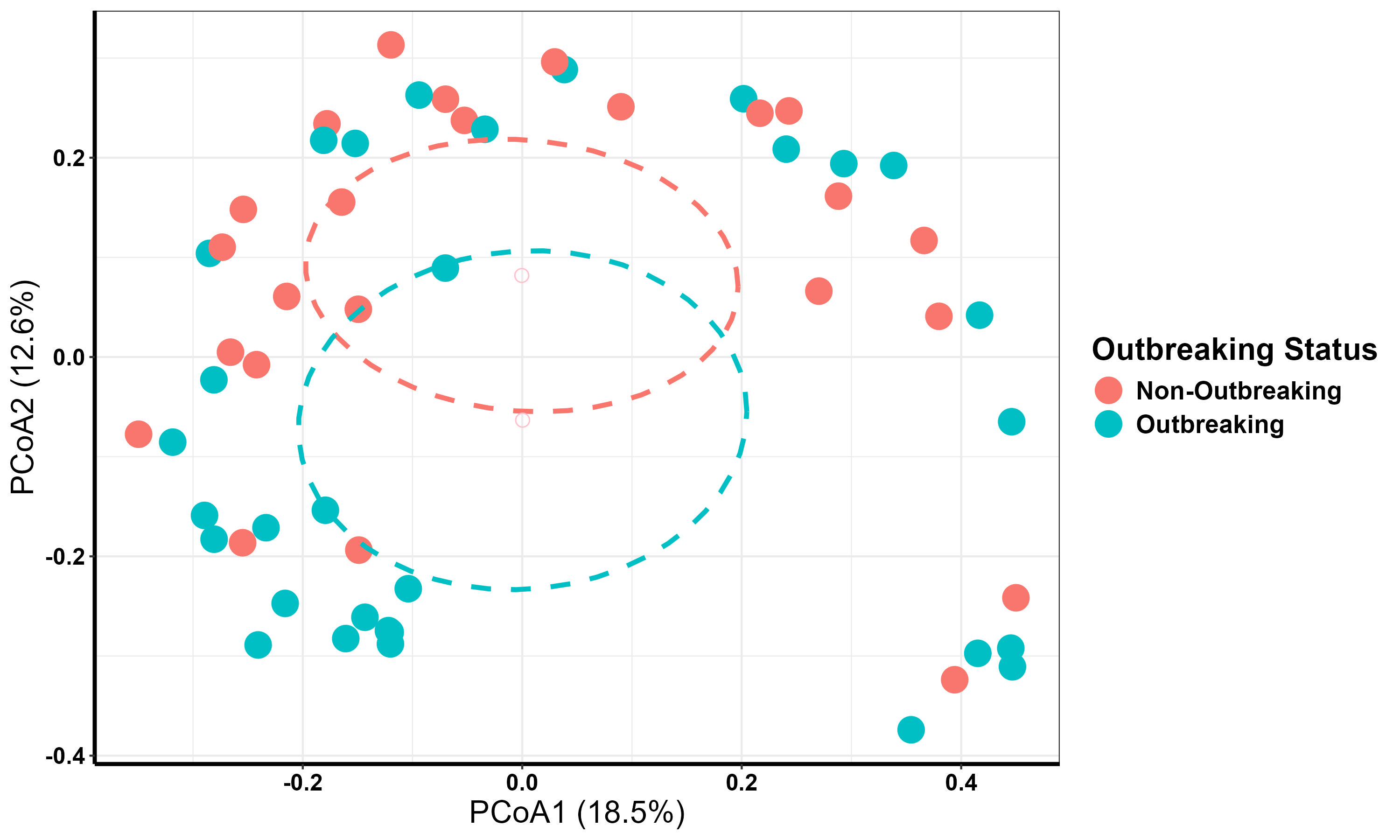

Supplement: SUPPLEMENTARY FIGURE S7 — Principal Coordinate Analysis (PCoA) based on Bray-Curtis distances, showing differences in meta-microbiome composition according to outbreak status. Each dot represents the meta-microbiome composition of a single whitefly sample. PCoA1 and PCoA2 explain 18.5% and 12.6% of the variation, respectively. [file Image_7.jpeg]
